# 

**Published:** 1904-04-15

**Authors:** 


### CONTENTS.

Care of the Oral Cavity during Pregnancy, *By C. H. Oakman, D.D.S.* 163
Suppurative Pericementitis, - *By A. B. Conklin, M.D.* 166
Adrenalin and Cocain for Local Anesthesia, *By Dr. Fritz Hartwig* 175
The "Northwest" and the "East" - - - - 183
Pressure Anesthesia, - *By R. B. Tuller, D.D.S.* 186
Aluminum, - *By E. Bumgardner, D.D.S.* 193
EDITORIAL, - - - - - - - - - 196
ANNOUNCEMENTS, - - - - - - - 199
OBITUARY, - - - - - - - - - 205
INDENTS, - - - - - - - - - 206

